# Spectroscopic Study of Solvent Effects on the Electronic Absorption Spectra of Flavone and 7-Hydroxyflavone in Neat and Binary Solvent Mixtures

**DOI:** 10.3390/ijms12128895

**Published:** 2011-12-05

**Authors:** Matias I. Sancho, Maria C. Almandoz, Sonia E. Blanco, Eduardo A. Castro

**Affiliations:** 1 Physical Chemistry Laboratory, Faculty of Chemistry, Biochemistry and Pharmacy, San Luis National University, 5700 San Luis, Argentina; E-Mails: misancho@unsl.edu.ar (M.I.S.); mcalman@unsl.edu.ar (M.C.A.); sblanco@unsl.edu.ar (S.E.B.); 2 Multidisciplinary Institute of Biological Investigations (IMIBIO-SL) CONICET, 5700 San Luis, Argentina; 3 INIFTA, Chemistry Department, Faculty of Exact Sciences, La Plata National University, 1900 Buenos Aires, Argentina

**Keywords:** flavones, solvatochromism, LSER, preferential solvation, TD-DFT calculations

## Abstract

The solvatochromic characteristics of flavone and 7-hydroxyflavone were investigated in neat and binary solvent mixtures. The spectral shifts of these solutes were correlated with the Kamlet and Taft parameters (α, β and π*) using linear solvation energy relationships. The multiparametric analysis indicates that both specific hydrogen bond donor ability and non-specific dipolar interactions of the solvents play an important role in absorption maxima of flavone in pure solvents. The hydrogen bond acceptor ability of the solvent was the main parameter affecting the absorption maxima of 7-hydroxyflavone. The simulated absorption spectra using a TD-DFT method were in good agreement with the experimental ones for both flavones. Index of preferential solvation was calculated as a function of solvent composition. Preferential solvation by ethanol was detected in cyclohexane-ethanol and acetonitrile-ethanol mixtures for flavone and in acetonitrile-ethanol mixtures for 7-hydroxyflavone. These results indicate that intermolecular hydrogen bonds between solute and solvent are responsible for the non-linear variation of the solvatochromic shifts on the mole fraction of ethanol in the analyzed binary mixtures.

## 1. Introduction

The study of solvent effects on the structure and spectroscopic behavior of a solute is essential for the development of solution chemistry [1–5]. The presence of specific and non-specific interaction between the solvent and the solute molecules are responsible for the change in the molecular geometry, electronic structure and dipolar moment of the solute. These solute-solvent interactions affect the solute’s electronic absorption spectrum and this phenomenon is referred to as solvatochromism [6]. Moreover, the behavior of a solute in a neat solvent is very different from the behavior in mixed binary solvent systems. In these kinds of systems, the solute may induce a change in the composition of the solvents in the cybotactic region compared to that in the bulk leading to preferential solvation. This situation commonly results from specific (hydrogen bonding) and non-specific (dielectric effects) interactions. The separation of specific from non-specific interactions in the interpretation of experimental measurements of absorption spectra is a difficult task. Quantitative measures for polarity are necessary in order to differentiate between these two effects [7]. Among all the existing solvent polarity scales, in this work we use the empirical solvatochromic scale of Kamlet and Taft [8,9]. With the purpose of analyzing solvent effects on a given solute, one of the most successful quantitative treatments is linear solvation energy relationships (LSER). This treatment uses a multiparameter equation of the form:

(1) $$XYZ={\mathrm{XYZ}}_{0}+s\pi *+a\alpha +b\beta$$

where *XYZ* is the solute property; *XYZ_0_* is the value of this property for the same solute in an hypothetical solvent for which π* = α = β = 0, π* is an index of the solvent dipolarity/polarizability, α is a measure of the solvent hydrogen-bond donor (HBD) capacity, β is a measure of the solvent hydrogen-bond acceptor (HBA) capacity and *s*, *a* and *b* are susceptibility constants.

Flavonoids are natural compounds present in fruits and vegetables and they exhibit a wide variety of pharmaceutical and biological properties [10–15]. Among flavonoids, flavones and particularly hydroxyflavones have been reported to show these important properties coupled with low toxicity. They also exhibit some interesting photophysical and photochemical properties [16,17]. These flavones have potential applications as highly sensitive environmental probes in micellar systems [18]. There have been a large number of studies on solvatochromism of different probe molecules like fluorenones, anthraquinones and luminol [19–21]. Nevertheless, the information on the solvatochromic behavior of flavones is rather scarce. For this reason, a systematic study of solvent effects on flavones is not only interesting but also necessary. In the present work, an experimental and theoretical study on the solvatochromic effects of flavone (**F**) and 7-hydroxyflavone (**7HF**) is carried out in pure solvents as well as in binary mixture solvents using UV-vis spectroscopy and DFT methods in order to describe the solute-solvent interactions that these compounds present.

## 2. Results and Discussion

### 2.1. Solvatochromism of **F** and **7HF** in Single Solvents

**Figure 1:**
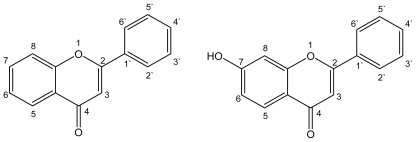
Structure and chemical numbering system of flavone (**left**) and 7-hydroxyflavone (**right**).

**Figure 2:**
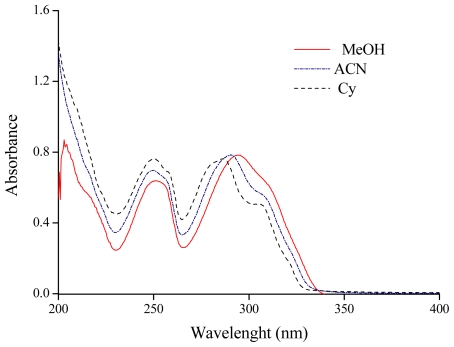
UV-visible absorption spectra of flavone in different solvents.

Figure 1 shows the structure and chemical numbering system of **F** and **7HF**. The UV-vis absorption spectra of **F** exhibit two absorbance maxima (band I and band II). Band I can be found in 286 to 295 nm range and band II next to 250 nm depending on the used solvent. The solvent exerts an influence on the electronic absorption, changing their shape, and spectral maxima positions. Figure 2 shows as example the electronic absorption spectra of **F** in three representative solvents, cyclohexane (Cy), acetonitrile (ACN) and methanol (MeOH). Some of the solvents employed, 1-butanol (1-BuOH), *N*,*N*-Dimethylformamide (DMF) and dimethylsulfoxide (DMSO), absorb radiation in the region of the spectra where **F** exhibits the higher energy band (band II). In addition, the solvatochromism is only observed in the lower energy band (band I) of the absorption spectrum. This band presents a bathochromic shift with increasing π* values of the solvents.

In Table 1 the maximum absorption wavelength (λ_max_) of the band I of **F** in neat solvents along with relevant solvents parameters [22] are summarized. A red shift on band I of 4.3 nm and 8.0 nm is observed upon going from Cy (α = 0, β = 0 and π* = 0) to ACN (α = 0.19, β = 0.40 and π* = 0.75) and from Cy to MeOH (α = 0.98, β = 0.66 and π* = 0.60) respectively. It is important to notice that the λ_max_ in DMSO is closer to the values obtained in polar protic solvents than the corresponding values registered in electron pair donating (EPD) solvents. In solvents with specific interactions (high values of α and β) there is no clear trend between the solvent polarity and λ_max_ values.

**Figure 3:**
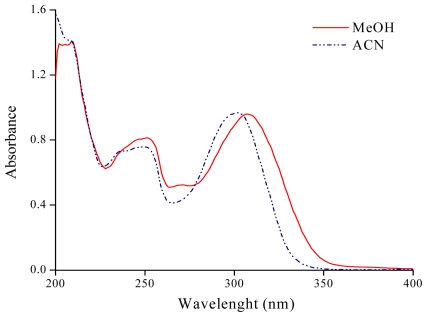
UV-visible absorption spectra of 7-hydroxyflavone in methanol and acetonitrile.

Figure 3 shows the characteristic electronic absorption spectra of **7HF** in ACN and MeOH. In Table 1 the λ_max_ values of the band I of **7HF** in pure solvents are summarized. For non-polar solvents **7HF** presents a very limited solubility and the UV-vis spectra were not recorded. In 1,4-dioxane (α = 0, β = 0.37 and π* = 0.55) band I is located at 300.0 nm and suffers a bathochromic shift with increasing π* values in EPD solvent. For this group of solvents, the maximum shift is observed in DMSO, where λ_max_ is at 308.1 nm. Band I is located between 308.4 nm and 310.1 nm in polar protic solvents and there is no clear trend between λ_max_ and the analyzed parameters of the solvents.

**Figure 4:**
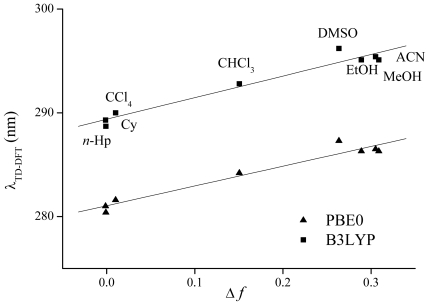
Correlation between the absorption wavelength of flavone calculated with the PCM/TD-DFT method and solvent polarity function Δ*f*.

**Figure 5:**
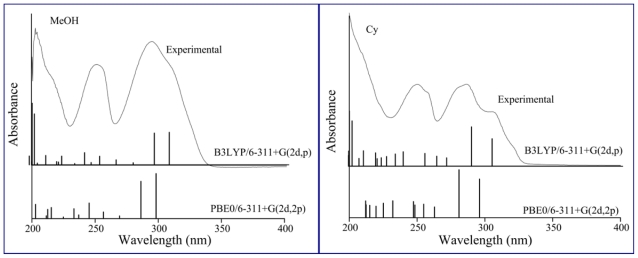
Experimental UV-visible and theoretical spectra of flavone in cyclohexane and methanol and positions of electronic transitions calculated with the TD-DFT method and the IEF-PCM model.

The electronic transitions were calculated using the TD-B3LYP/6-311+G(2d,p) and TD-PBE0/6-311+G(2d,2p) methods. The values of the calculated absorption wavelengths (λ_TD-DFT_), oscillator strength (*f*) and the Molecular Orbitals (MOs) involved in the main transitions are reported in Table 2. The calculated (λ_TD-DFT_) are found to be associated with solvent polarity function Δ*f*. This parameter can be calculated as **Δ***f* = (ɛ − 1)/(2ɛ + 1) − (*n*^2^ − 1)/(2*n*^2^ + 1), where ɛ is the dielectric constant and *n* is the refractive index. For the calculation of this parameter the ɛ reported in Table 1 and the *n* values taken from tabulated data [23] were used. A good linear relation between λ_B3LYP_ and λ_PBE0_ with **Δ***f* is found and shown in Figure 4. It can be observed from this figure that the λ_TD-DFT_ in DMSO presents a remarkable deviation from linearity. Zhao *et al*. have studied the solvatochromism of Coumarin 102 by means of TD-DFT methods and they observed the same deviation in DMSO [24]. These authors attribute this anomaly to strong long-range bulk electrostatic effects and a very large π* value of DMSO. In Figure 5 the simulated and experimental UV-vis spectra of **F** are shown in Cy and MeOH. It can be seen from this figure that the experimental spectra are well reproduced by the simulated ones. The maximum absorption wavelength of band I is predicted at λ_B3LYP_ = 289.3 nm and λ_PBE0_ = 281.0 nm (λ_exp_ = 286.1 nm) in Cy and λ_B3LYP_ = 295.1 nm and λ_PBE0_ = 286.3 nm (λ_exp_ = 294.1 nm) in MeOH. The shoulder is predicted at λ_B3LYP_ = 304.4 nm and λ_PBE0_ = 296.1 nm (λ_exp_ = 305.02 nm) in Cy and λ_B3LYP_ = 306.8 nm and λ_PBE0_ = 298.4 nm (λ_exp_ ≈ 307 nm) in MeOH. The results reported in Table 2 indicate that B3LYP overestimate the λ_exp_ while PBE0 underestimate them. The mean absolute error (MAE) of the λ_TD-DFT_ calculated with the B3LYP is of 2.1 nm whereas for PBE0 the MAE is 6.7 nm. On the basis of the agreement with λ_exp_ values, the B3LYP would be the functional of choice to calculate the absorption wavelengths of **F**. A statistical analysis using simple linear regression between the λ_TD-DFT_ and the λ_exp_ values does not improve the theoretical results.

**Figure 6:**
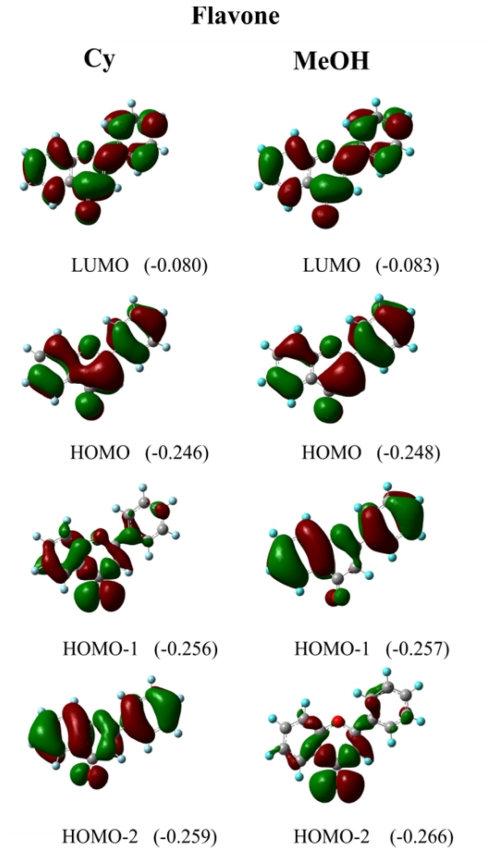
Molecular orbitals involved in the electronic transitions of flavone in cyclohexane and methanol calculated at the B3LYP/6-311+G(2d,p) level. The energy values of each molecular orbital are in parenthesis.

The TD-B3LYP results indicate that for the band I of **F** the MOs responsible for the electronic transition are HOMO-2 → LUMO with a ππ* character. This is only observed in Cy, *n*-Hp and CCl_4_. In solvents with specific solute–solvent interactions the transition is HOMO-1 → LUMO presenting also a ππ* character. In Figure 6 the shape of the MOs involved in the electronic transitions in Cy and MeOH is depicted. This figure shows that the electronic density on the HOMO-1 is delocalized all over the molecular structure in MeOH whereas in Cy the electronic density is more localized on the carbonylic oxygen atom. The opposite phenomenon is observed for HOMO-2. In MeOH the electronic density is localized on the carbonylic oxygen and in Cy it is delocalized over the whole molecule. This result is consistent with the red shift observed in **F** upon going from non-polar to polar protic solvents. The HOMO-2 → LUMO transition posses a higher energy than the HOMO-1 → LUMO, and therefore the absorption band is shifted to higher wavelengths in solvents with specific interactions. Similar results are observed with MOs calculated with the TD-PBE0 method. The only difference with the TD-B3LYP results is that the MOs responsible for the electronic transition are HOMO-1 → LUMO for all the analyzed solvents. The ππ* character of this transition is also reproduced by the TD-PBE0 method. All the MOs of **F** in the analyzed solvent are shown in the supplementary material (Figures S1 and S3).

**Figure 7:**
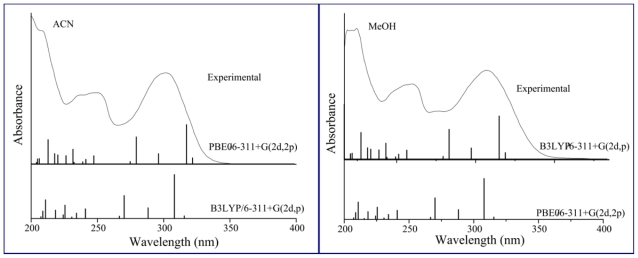
Experimental UV-visible spectra of 7-hydroxyflavone in acetonitrile and methanol and positions of electronic transitions calculated with the TD-DFT method and the IEF-PCM model.

Figure 7 shows the simulated and experimental spectra of **7HF** in ACN and MeOH. A fairly good agreement between the experimental and the calculated absorption wavelengths is also observed. For example, the maximum absorption wavelength of band I is predicted at λ_B3LYP_ = 318.1 nm and λ_PBE0_ = 308.6 nm (λ_exp_ = 301.5 nm) in ACN and λ_B3LYP_ = 318.0 nm and λ_PBE0_ = 308.0 nm (λ_exp_ = 308.4 nm) in MeOH. No statistical analysis can be made here since there are only four solvents to compare λ_TD-DFT_ values with λ_exp_ values. However, on the basis of the agreement between λ_exp_ with λ_TD-DFT_, the PBE0 would be the functional of choice to calculate the absorption wavelengths of **7HF**. A good linear relation between λ_B3LYP_ and λ_PBE0_ with Δ*f* is also found and the same anomaly observed for **F** in DMSO is detected for **7FH**.

**Figure 8:**
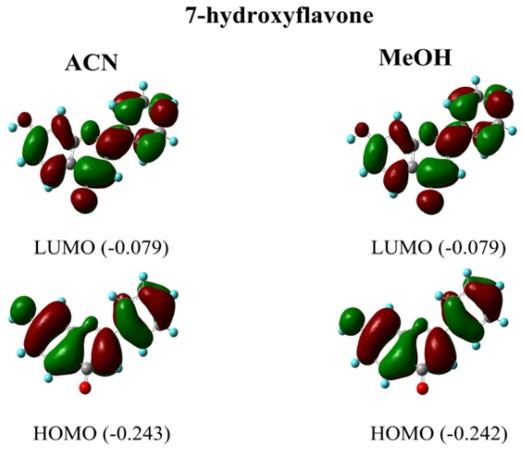
Molecular orbitals involved in the electronic transitions of 7-hydroxyflavone in acetonitrile and methanol calculated at the B3LYP/6-311+G(2d,p) level. The energy values of each molecular orbital are in parenthesis.

Lapouge and Cornard have simulated the electronic absorption spectra of some hydroxylated flavones; 3-hydroxyflavone (**3HF**) and 5-hydroxyflavone (**5HF**) by means of a TD-DFT method similar to the one used in this work [25]. These authors report a HOMO to LUMO transition in MeOH for band I of **3HF** and **5HF**, with an electronic redistribution over the molecule for **3HF** and a charge transfer from the chromone moiety to the B ring for **5HF**. Our results indicate that in the case of **7HF** the transition is HOMO → LUMO with a ππ* character in all the analyzed solvents. Figure 8 illustrates the shape of the MOs involved in the electronic transitions of **7HF** in ACN and MeOH. It can be seen that there is no charge transfer; the transition corresponds to an electronic redistribution over the whole molecule. No significant changes are observed between TD-B3LYP and TD-PBE0 results. All the MOs of **7HF** in the analyzed solvent are also shown in the supplementary material (Figures S2 and S4).

The frequency analysis showed that **7HF** have more vibrational modes than **F** due to the presence of the OH group. It is important to notice that the UV-vis absorption spectra were calculated from vertical transitions between electronic states of the ground states, without taking vibrations into account. Moreover, the calculated excitation energies indicate that the transition for **F** presents a higher energy (lower absorption wavelength) than the corresponding to **7HF**. These results are in good agreement with the experimental measurements.

The PCM model used in the DFT calculations takes into account the dielectric constant and the refractive index of each solvent. These parameters describe the solvent fairly well, but they are not adequate to characterize specific solute–solvent interactions. For this reason, in order to obtain a better description on the solvatochromism of **F** and **7HF**, the empirical solvation parameters of Kamlet and Taft were analyzed. Table 1 summarizes the corresponding parameters α, β and π* of the used solvents. The maximum absorption wavenumber (ν̄_max_) can be related to these parameters separately. However, the use of a multiparametric equation provides a better quantitative description of the solvatochromic shifts and takes into account specific (α and β) and non-specific (π*) interactions. The following multiparametric relationship was obtained for **F** applying Equation (1) and using the ν̄_max_ values listed in Table 1:

(2) $$\begin{array}{c} {\bar{\nu }}_{\text{max}}\;({\text{cm}}^{-1})=(34.88\times 10^{3}\pm 78)-(650\pm 165)\;\pi^{*}-(573\pm 127)\;\alpha -(149\pm 178)\;\beta \\ \left(n=14,R^{2}=0.9063,\text{SD}=132,Fisher’s\;F=32.23,p-\text{value}<0.0001\right) \end{array}$$

The relative contributions of the parameters are: π*-47.3%, α-41.7% and β-10.9%. The selected variables explain 90.6% of the variability of ν̄_max_ in different solvents. It must be noticed that the standard error of the β term indicates that there is not a statistically significant variable in the regression equation and this parameter may be neglected. This is a reasonable result because **F** is incapable of HBD abilities. Equation (2) reveals that the solvation of **F** is mainly determined by dipolar interactions (π*) and the donation of hydrogen bonds (α) by the solvent molecules. For this reason the multiparametric equation can be expressed as a biparametric equation,

(3) $$\begin{array}{c} {\bar{\nu }}_{\text{max}}\;({\text{cm}}^{-1})=(34.88\times 10^{3}\pm 77)-(740\pm 123)\;\pi^{*}-(650\pm 88)\;\alpha \\ \left(n=14,R^{2}=0.8996,\text{SD}=130,Fisher’s\;F=49.31,p-\text{value}<0.0001\right) \end{array}$$

The relative contributions of both parameters are similar (π*-53.3%, α-46.7%). Moreover, the negative signs of the π* and α coefficients indicate that the specific and non-specific interactions in protic solvents may stabilize the excited state more than the ground state, resulting in bathochromic shift. Taking into account the contribution of non-specific interactions, the previously mentioned anomalous ν̄_max_ value in DMSO could be explained. The π* parameter of this solvent is considerably higher than the corresponding values of the polar protic solvents.

When Equation (1) is used to analyze the solvatochromism of **7HF**, the following result is obtained:

(4) $$\begin{array}{c} {\bar{\nu }}_{\text{max}}\;({\text{cm}}^{-1})=(34.13\times 10^{3}\pm 232)-(428\pm 287)\;\pi^{*}-(379\pm 162)\;\alpha -(1533\pm 258)\;\beta \\ \left(n=10,R^{2}=0.9467,\text{SD}=105,Fisher’s\;F=35.57,p-\text{value}<0.0003\right) \end{array}$$

The relative contributions of the parameters are: π*-18.3%, α-16.2% and β-65.5%. The selected variables explain 94.67% of the variability of ν̄_max_ in different solvents. It can be observed that specific interactions have the main contribution to the solvatochromism of **7HF**. For this reason Equation (4) can be presented as,

(5) $$\begin{array}{c} {\bar{\nu }}_{\text{max}}\;({\text{cm}}^{-1})=(34.86\times 10^{3}\pm 159)-(195\pm 114)\;\alpha -(1666\pm 262)\;\beta \\ \left(n=10,R^{2}=0.9271,\text{SD}=114,Fisher’s\;F=44.51,p-\text{value}<0.0001\right) \end{array}$$

The relative contributions of the parameters now are: α-10.5% and β-89.5%. Various types of intermolecular hydrogen bonding (IHBs) may affect the electronic transitions of this compound. However, the results obtained suggest that the HBA ability of the solvent is the most important factor to explain the solvatochromism. The negative sign of β is consistent with the bathochromic shift observed in solvents with higher β values, indicating that the hydrogen bonds formed by the OH group of **7HF** with the solvent may stabilize the excited state more than the ground state. It is important to notice that the β parameter of the DMSO is comparable with the corresponding values of the polar protic solvents. This could be the reason for the observed spectral shifts. For example, ν̄_max_ = 34.01 × 10^3^ cm^−1^ in DMSO and in EtOH.

To summarize, the solvatochromic shifts of **F** can be explained by the polarization effects of the solvents and by the formation of IHBs in the oxygen atom of the carbonyl of **F** in protic solvents. These interactions stabilize the excited state more than the ground state, and a bathochromic shift is observed in solvents with higher π* and α parameters. Conversely, the solvatochromic shifts of **7HF** are mainly due to the formation of IHBs between the H in the hydroxyl group of **7HF** and the solvents. This interaction stabilizes the excited state more than the ground state, and a bathochromic shift is observed in solvents with a higher β parameter.

### 2.2. Solvatochromism of **F** and **7HF** in Binary Solvent Mixtures

The solvent effects on the electronic absorption spectra of **F** and **7HF** in binary mixtures were also analyzed. If the binary mixture is considered as an ideal one, ν̄_max_ of the solute should follow a linear additive model according to the following equation [26].

(6) $${\bar{\nu }}_{12\;\text{ideal}}={\bar{\nu }}_{1}\;X_{1}+{\bar{\nu }}_{2}\;X_{2}$$

In this equation *X*_1_ and *X*_2_ are the mole fraction of solvents 1 and 2, and ν̄_1_, ν̄_2_, ν̄_12_ are the values of ν̄_max_ of the studied flavonoids in solvent 1, solvent 2 and in the binary mixture, respectively.

**Figure 9:**
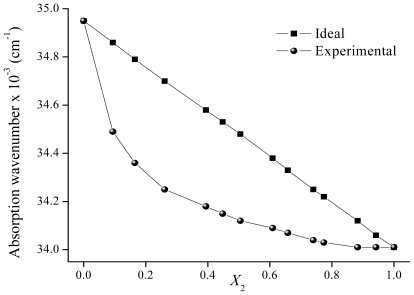
Maximum absorption wavenumber of flavone in Cy-EtOH mixtures determined for different mole fractions of EtOH.

**Figure 10:**
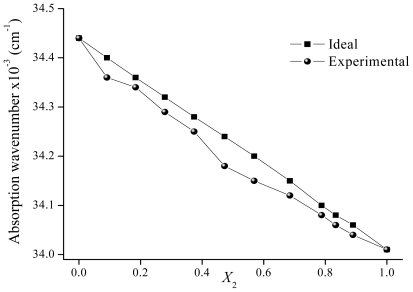
Maximum absorption wavenumber of flavone in ACN-EtOH mixtures determined for different mole fractions of EtOH.

**Figure 11:**
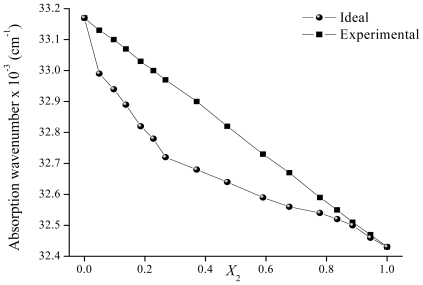
Maximum absorption wavenumber of 7-hydroxyflavone in ACN-EtOH mixtures determined for different mole fractions of EtOH.

The ν̄_12 ideal_ values in the mixtures can be calculated by using Equation (6) over the entire range of solvent composition. The calculated (ν̄_12 ideal_) and experimental (ν̄_12_) values for binary mixtures (Cy-EtOH and ACN-EtOH) are plotted (Figures 9–11) against the bulk mole fraction of EtOH (*X*_2_). As can be observed, the experimental values deviate from the linearity and the curvature of the plot indicates that the solute is preferentially solvated by one of the solvents. In order to analyze the interactions observed, the preferential solvation approach [27] can be used. This approach considers the solvent to be distributed between two phases, the bulk and the solvation shell of the solute. It is assumed that the solvation shell is made up of independent sites that are always occupied. In a non-ideal mixture, the ν̄_12_ can be expressed by Equation (7)

(7) $${\bar{\nu }}_{12}={\bar{\nu }}_{1}\;X_{1}^{L}+{\bar{\nu }}_{2}\;X_{2}^{L}$$

where *X*_1_*^L^* and *X*_2_*^L^* represent the mole fraction of the solvents 1 and 2 in the solvation shell of the solute, respectively. *X*_2_*^L^* can be calculated from experimental measurements through the following expression:

(8) $$X_{2}^{L}=\frac{{\bar{\nu }}_{12}-{\bar{\nu }}_{1}}{{\bar{\nu }}_{2}-{\bar{\nu }}_{1}}$$

In order to quantify the extent of preferential solvation, a parameter δ_S2_ may be used. This parameter can be defined as the difference between *X*_2_*^L^* and *X*_2_ [28]

(9) $$\delta_{s2}=X_{2}^{L}-X_{2}$$

A positive value of δ_S2_ indicates a preference for solvent 2 over solvent 1, while a negative value of δ_S2_ signifies the opposite.

The maximum absorption wavenumber of **F** and **7HF** were measured in two binary mixtures, Cy-EtOH and ACN-EtOH, using different solvent ratios. In the case of **F**, there is a decrease in the ν̄_12_ values as the mole fraction of EtOH increases in the mixtures. Figure 9 shows the variation of ν̄_12_ as a function of *X*_2_. As can be seen from this figure, the decrease of ν̄_12_ is more prominent in Cy rich regions. The preferential solvation is clearly observed and the index δ_S2_ for EtOH is maximum at *X*_2_= = 0.26. This means that at low concentrations of EtOH in the bulk phase, there are more EtOH than Cy molecules surrounding the solvation shell of **F**. In order to rationalize this effect, it must be noticed that our results for pure solvents indicate that the solvatochromism of **F** is mainly affected by the HBD ability of the solvent (see Section 2.1.). Then, the IHB formation between **F** and EtOH may be responsible for the preferential solvation of this solute in Cy-EtOH mixtures. In addition, the π* parameter of EtOH (0.54) is higher than the one corresponding to Cy (0.00), and the non-specific dipolar interactions may also contribute to the preferential solvation of **F** by EtOH.

Different observations can be made when Cy (solvent with non-specific interactions) is replaced by ACN (EPD solvent) in the binary mixture. In Figure 10 the variation of ν̄_12_ with *X_2_* in ACN-EtOH mixtures is depicted. As EtOH is added in the mixture a decrease in the ν̄_12_ values is also observed, but the deviations from linearity are smaller than in Cy-EtOH. Preferential solvation is detected and is almost constant in all the *X_2_* range. The index δ_S2_ for EtOH is maximum at *X_2_* = 0.47. In Table 3 the values of δ_S2_ for **F** in both analyzed mixtures are listed. The numerical values of δ_S2_ are smaller when ACN is used as co-solvent than when Cy is the co-solvent. Thus, it can be concluded that the preferential solvation by EtOH is more clearly observed in Cy-EtOH than in ACN-EtOH mixtures. These results may be explained in terms of the α and π* parameters of the solvents. Cy is a non-polar solvent with no HBD ability (α = 0) and a very low polarization capacity (π* = 0). ACN has no HBD ability either (α = 0.19) but it presents a notable polarizability (π* = 0.75) due to the electron pair of the nitrogen atom. Then, ACN may present non-specific interactions with this solute.

The analysis for **7HF** was made only in ACN-EtOH mixtures due to the very low solubility of the compound in pure Cy. Figure 11 shows the variation of **ν̄_12_** with *X_2_*. Preferential solvation by EtOH is clearly observed from this figure and the index δ_S2_ for EtOH is maximum at *X_2_* = 0.27 (Table 4). This means that at lower concentrations of EtOH in the bulk phase, the solvation shell of **7HF** presents a higher concentration of this solvent. EtOH may form IHBs through the OH group or the carbonyl group of this solute, and these interactions may be responsible for the phenomenon observed. For *X_2_* > 0.80, the preferential solvation notably decreases, indicating that the solvation of **7HF** in the mixture is close to the ideal behavior. In the EtOH rich regions there is always a possibility of self association through hydrogen bonding and this competes with solute–solvent interactions [29]. Then, a molecule of EtOH will be relatively preferred by other EtOH molecules rather than **7HF** molecules.

## 3. Experimental Section

### 3.1. Chemicals and Reagents

Flavone and 7-hydroxyflavone were purchased from Sigma-Aldrich Chemical Co. and they were purified by recrystallization from methanol-water. The purity control was performed determining its chromatographic properties (TLC and HPLC). The solvent employed, Cy (≥99.9%), *n*-Hp (≥99.3%), CCl_4_ (≥99.9%), 1,4-dioxane (≥99.9%), ACN (≥99.8%), DMF (≥99.9%), DMSO (≥99.8%), CHCl_3_(≥99.8%), 1-Oc (≥99.0%), 1-Bu (≥99.9%), 2-Pr (≥99.9%), 1-Pr (≥99.8%), EtOH (≥99.9%) and MeOH (≥99.9%) from Merck KGaA (Germany) were all HPLC or spectroscopic grade and were used without further purification.

### 3.2. Procedures

The solutions of **F** and **7HF** were prepared with a concentration of 4.1 × 10^−5^ M in pure solvents. Binary mixtures of Cy-EtOH and ACN-EtOH were prepared from the corresponding solutions by mixing them in the following ratios: 1:9, 2:8, 3:7, 4:6, 5:5, 6:4, 7:3, 8:2 and 9:1. All the solutions were prepared by weight with an accuracy of ±0.0001 g and were stabilized at 25.0 ± 0.1 °C for 10 min. Then their spectra were recorded in a Cary 50-Varian spectrophotometer with thermostatizable cells of 1 cm optical path in the 200–400 nm interval. All spectra were corrected for solvent background by calibrating the instrument to the blank solvent and the experiments were carried out in duplicate.

## 4. Computational Details

The initial molecular geometries of **F** and **7HF** were optimized with the GAUSSIAN 03 [30] programs package using the hybrid DFT functional B3LYP [31,32] at 6-311G+(2d,p) level. To analyze the solvent effects on the optimized structures in gas-phase the Polarizable Continuum model with the integral equation formalism (IEF-PCM) [33] was used, and the UA0 standard radii were employed to build the molecular cavity. The corresponding frequencies were calculated to make sure that the structures obtained were true minima. The dielectric constants used in the PCM calculation are the default values in GAUSSIAN 03 and they are listed in Table 1. Relevant structural parameters of the gas and solution phase optimized flavonoids are reported in the Supplementary File (Table S1). Using the minimum energy structures of **F** and **7HF** as starting points, vertical excitation energies and the corresponding absorption wavelengths were calculated within the non-equilibrium time-dependent (TD-DFT) framework [34]. For this aim, two hybrid functionals have been used: B3LYP and PBE0 [35,36] in combination with the PCM method. The PBE0 does not contain any adjustable parameter fitted to specific property, and recent studied on coumarins suggests that provides more reliable results compared with experimental results [24,37,38]. To simulate the UV-visible spectra only the excitation energies with oscillator strength (*f*) higher than 0.01 were used.

## 5. Conclusions

The solvent effect on the electronic absorption spectra of two flavonoids (**F** and **7HF**) was analyzed in neat and binary solvent mixtures of diverse nature using UV-vis spectroscopy and DFT calculations. The solvatochromic shifts observed in pure solvent were evaluated using linear solvation energy relationships (LSER) with the Kamlet and Taft parameters, and the electronic transitions were explained with the PCM/TD-DFT treatment. The spectroscopic behavior in binary solvent mixtures was analyzed using the preferential solvation approach. The solvatochromism of **F** in pure solvent is affected mainly by the HBD abilities and the dipolarity/polarizability of the solvent. The electronic transitions of band I calculated with the B3LYP are HOMO-1 → LUMO in solvents with specific interactions and HOMO-2 → LUMO in solvents without specific interactions with a ππ* character in all the cases. On the other hand, the solvatochromic shifts of **7HF** are affected primarily by the HBA abilities of the solvent. The electronic transitions of band I are HOMO → LUMO with a ππ* character in all the analyzed solvents. In Cy-EtOH solvent mixtures, **F** exhibits preferential solvation by EtOH in the whole concentration range, this effect being more marked in Cy rich regions. In ACN-EtOH mixtures preferential solvation by EtOH is also observed in the whole concentration range. However, the indexes of preferential solvation are smaller than the ones obtained for Cy-EtOH mixtures. Moreover, **7HF** also appears to be solvated preferentially by EtOH in ACN-EtOH mixtures at high concentrations of ACN in the bulk phase. At high EtOH concentrations in the bulk phase, preferential solvation is practically not observed.

## Supplementary Material



## Figures and Tables

**Table 1:** Absorption maxima of flavone and 7-hydroxyflavone in pure solvents and relevant solvent parameters. λ values are expressed in nm and ν̄values are in cm^−1^.

	Flavone		7(OH)Flavone					

Solvent	λ	λ× 10^−3^	λ	ν̄× 10^−3^	ɛ	π*	α	β
Cyclohexane (Cy)	286.1	34.95	NS		2.023	0.00	0.00	0.00
*n*-Heptane (*n*-Hp)	286.0	34.97	NS		1.920	−0.08	0.00	0.00
Carbon tetrachloride (CCl_4_)	289.0	34.60	NS		2.228	0.28	0.00	0.10
1,4-Dioxane	290.1	34.47	300.0	33.33	2.219	0.55	0.00	0.37
Acetonitrile (ACN)	290.4	34.44	301.5	33.17	36.64	0.75	0.19	0.40
*N*,*N*-Dimethylformamide (DMF)	292.0	34.25	305.6	32.72	38.25	0.88	0.00	0.69
Dimethylsulfoxide (DMSO)	294.0	34.01	308.1	32.46	46.70	1.00	0.00	0.76
Chloroform (CHCl_3_)	293.6	34.06	NS		4.900	0.58	0.44	0.00
1-Octanol (1-Oc)	294.9	33.91	310.1	32.25	10.30	0.40	0.77	0.81
1-Butanol (1-Bu)	295.0	33.90	309.4	32.32	17.84	0.47	0.84	0.84
2-Propanol (2-Pr)	294.0	34.01	308.5	32.41	20.18	0.48	0.76	0.84
1-Propanol (1-Pr)	295.1	33.89	309.1	32.35	20.80	0.52	0.84	0.90
Ethanol (EtOH)	294.0	34.01	308.4	32.43	24.55	0.54	0.86	0.75
Methanol (MeOH)	294.1	34.00	308.4	32.43	32.63	0.60	0.98	0.66

NS: the compound presents a limited solubility in the indicated solvent.

**Table 2:** Calculated (λ_TD-DFT_) and experimental (λ_exp_) wavelengths of Band I of flavone and 7-hydroxyflavone, the Molecular Orbitals (MOs) involved in the electronic transitions in different solvents and oscillator strength (*f*).

Flavone							

	B3LYP			PBE0			
	
Solvent	λ_TD-DFT_	*f*	MOs	λ_TD-DFT_	*f*	MOs	λ_exp_
Cy	289.3	0.469	H-2 → L (79%)	281.0	0.452	H-1 → L (48%)	286.1
*n*-Hp	288.7	0.470	H-2 → L (82%)	280.4	0.453	H-1 → L (55%)	286.0
CCl_4_	290.0	0.465	H-2 → L (73%)	281.6	0.448	H-1 → L (55%)	289.0
ACN	295.4	0.359	H-1 → L (87%)	286.5	0.346	H-1 → L (87%)	290.4
DMSO	296.2	0.355	H-1 → L (88%)	287.3	0.345	H-1 → L (88%)	294.0
CHCl_3_	292.8	0.415	H-1 → L (83%)	284.2	0.401	H-1 → L (85%)	293.6
EtOH	295.1	0.364	H-1 → L (87%)	286.3	0.351	H-1 → L (87%)	294.0
MeOH	295.1	0.359	H-1 → L (87%)	286.3	0.346	H-1 → L (87%)	294.1
**7(OH)Flavone**							

	**B3LYP**			**PBE0**			
	
**Solvent**	**λ_TD-DFT_**	***f***	**MOs**	**λ_TD-DFT_**	***f***	**MOs**	**λ_exp_**

Cy	309.5	0.462	H → L (76%)	299.8	0.520	H → L (83%)	NS
*n*-Hp	308.9	0.450	H → L (74%)	299.2	0.508	H → L (80%)	NS
CCl_4_	310.4	0.472	H → L (79%)	300.6	0.530	H → L (84%)	NS
ACN	318.1	0.395	H → L (78%)	308.6	0.478	H → L (82%)	301.5
DMSO	318.9	0.378	H → L (70%)	309.1	0.496	H → L (85%)	308.1
CHCl_3_	314.3	0.468	H → L (86%)	304.4	0.524	H → L (88%)	NS
EtOH	317.7	0.411	H → L (80%)	307.7	0.485	H → L (86%)	308.4
MeOH	318.0	0.392	H → L (78%)	308.0	0.474	H → L (85%)	308.4

NS: the compound presents a limited solubility in the indicated solvent; H and L represent the HOMO and LUMO respectively.

**Table 3:** Indexes of preferential solvation (δ_S2_) of flavone in binary solvent mixtures. *X*_2_ is the ethanol mole fraction in the mixture, ν̄_12_ is the experimental absorption wavenumber measured in the mixture and ν̄_12 ideal_ are calculated by using Equation (6). ν̄_12_ and ν̄_12 ideal_ are expressed in cm^−1^.

Cy-EtOH				ACN-EtOH			

*X*_2_	ν̄_12_ × 10^−3^	ν̄_12 ideal_ × 10^−3^	δ_S2_	*X*_2_	ν̄_12_× 10^−3^	ν̄_12 ideal_ × 10^−3^	δ_S2_
0.000	34.95	34.95		0.000	34.44	34.44	
0.094	34.49	34.86	0.395	0.091	34.36	34.40	0.095
0.165	34.36	34.79	0.463	0.184	34.34	34.36	0.049
0.261	34.25	34.70	0.484	0.279	34.29	34.32	0.070
0.394	34.18	34.58	0.425	0.374	34.25	34.28	0.068
0.448	34.15	34.53	0.403	0.473	34.18	34.24	0.132
0.505	34.12	34.48	0.378	0.569	34.15	34.20	0.105
0.609	34.09	34.38	0.306	0.685	34.12	34.15	0.059
0.658	34.07	34.33	0.278	0.788	34.08	34.10	0.049
0.740	34.04	34.25	0.228	0.834	34.06	34.08	0.050
0.774	34.03	34.22	0.205	0.890	34.04	34.06	0.040
0.883	34.01	34.12	0.117	1.000	34.01	34.01	
0.942	34.01	34.06	0.058				
1.000	34.01	34.01					

**Table 4:** Indexes of preferential solvation (δ_S2_) of 7-hydroxyflavone in ACN-EtOH mixture. *X*_2_ is the ethanol mole fraction in the mixture, ν̄_12_ is the experimental absorption wavenumber measured in the mixture and ν̄_12 ideal_ are calculated by using Equation (6). ν̄_12_ and ν̄_12 ideal_ are expressed in cm^−1^.

ACN-EtOH			

*X*_2_	ν̄_12_ × 10^−3^	ν̄_12 ideal_ × 10^−3^	δ_S2_
0.000	33.17	33.17	
0.049	32.99	33.13	0.194
0.097	32.94	33.10	0.214
0.137	32.89	33.07	0.241
0.186	32.82	33.03	0.287
0.228	32.78	33.00	0.299
0.268	32.72	32.97	0.340
0.371	32.68	32.90	0.291
0.472	32.64	32.82	0.244
0.590	32.59	32.73	0.194
0.677	32.56	32.67	0.147
0.778	32.54	32.59	0.073
0.835	32.52	32.55	0.043
0.886	32.50	32.51	0.019
0.945	32.46	32.47	0.014
1.000	32.43	32.43	
