# Supplementary material for: Spectroscopic Study of Solvent Effects on the Electronic Absorption Spectra of Flavone and 7-Hydroxyflavone in Neat and Binary Solvent Mixtures
Source: Int J Mol Sci. 2011 Dec 5;12(12):8895–912. doi: 10.3390/ijms12128895 (PMC3257107; doi:10.3390/ijms12128895)

Table S1. Relevant geometrical paramaters of flavone and 7(OH)flavone in gas and solution phase calculated at B3LYP/6-311+G(2d,p) theoretical level. Interatomic distances, *r*, in Å; bond angles and dihedral angles, *A* and ω, in degrees.

	**Flavone**		**7(OH)Flavone**				
	r **(C_4=O)**	**ω ($O_1C_2C_1C_2$)**	***r*** **(C_4=O)**	**ω ($O_1C_2C_1C_2$)**	***r*** **(C_7-O)**	***r*** **(O-H)**	***A*** **(C_7OH)**
gas	1.226	20.9	1.228	21.7	1.363	0.964	109.8
Cy	1.231	21.4	1.233	22.1	1.360	0.970	110.5
n-Hp	1.231	21.5	1.232	22.2	1.360	0.970	110.5
CCl_4	1.232	21.4	1.233	22.0	1.360	0.971	110.6
ACN	1.240	20.4	1.242	21.8	1.354	0.985	111.3
DMSO	1.240	20.8	1.242	22.1	1.354	0.985	111.3
$CHCl_3$	1.236	21.3	1.238	22.2	1.357	0.977	111.0
EtOH	1.240	20.8	1.242	22.0	1.355	0.984	111.2
MeOH	1.240	20.7	1.242	21.6	1.355	0.985	111.2

Figure S1. Computer Visualization of the Molecular Orbitals Involved in the Electronic Transitions of Flavone in Different Solvents Calculated with the TD-B3LYP/6-311+G(2d,p) Method and the IEF-PCM Model.

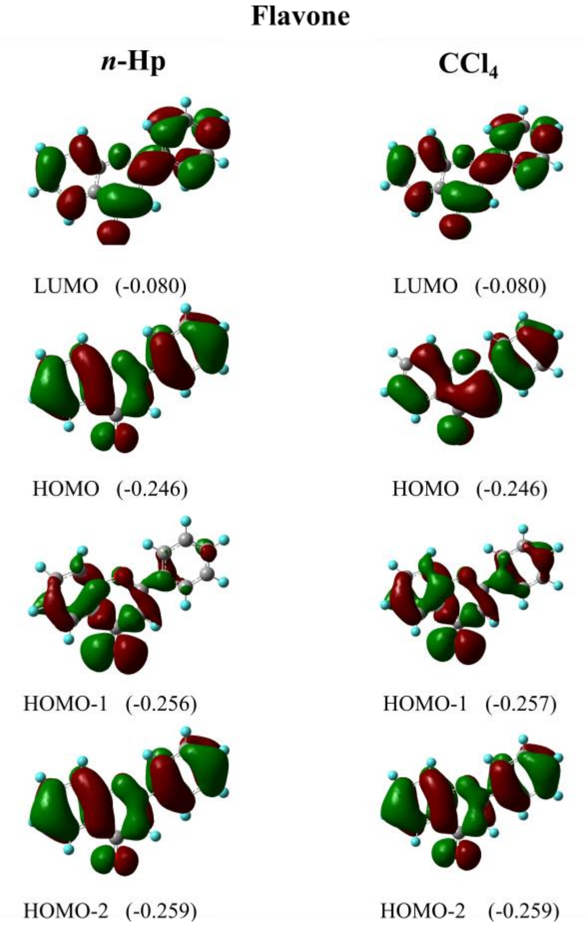

Figure S1. *Cont.*

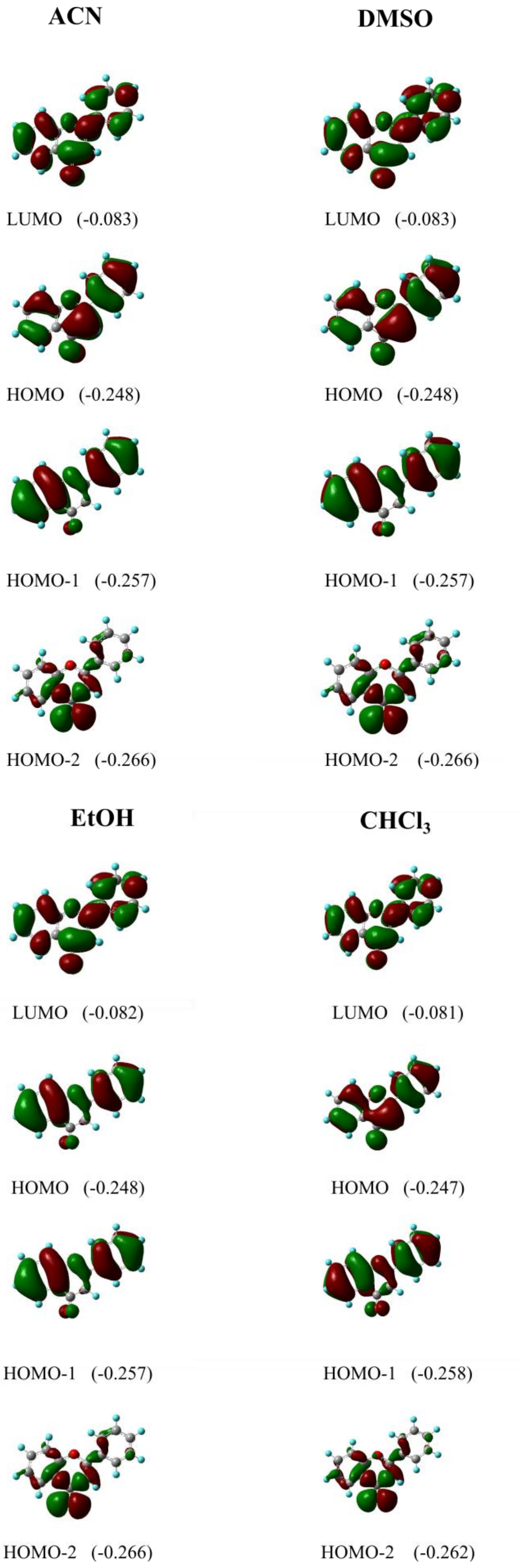

Figure S2. Computer Visualization of the Molecular Orbitals Involved in the Electronic Transitions of 7-hydroxyflavone in Different Solvents Calculated with the TD-B3LYP/6-311+G(2d,p) Method and the IEF-PCM Model.

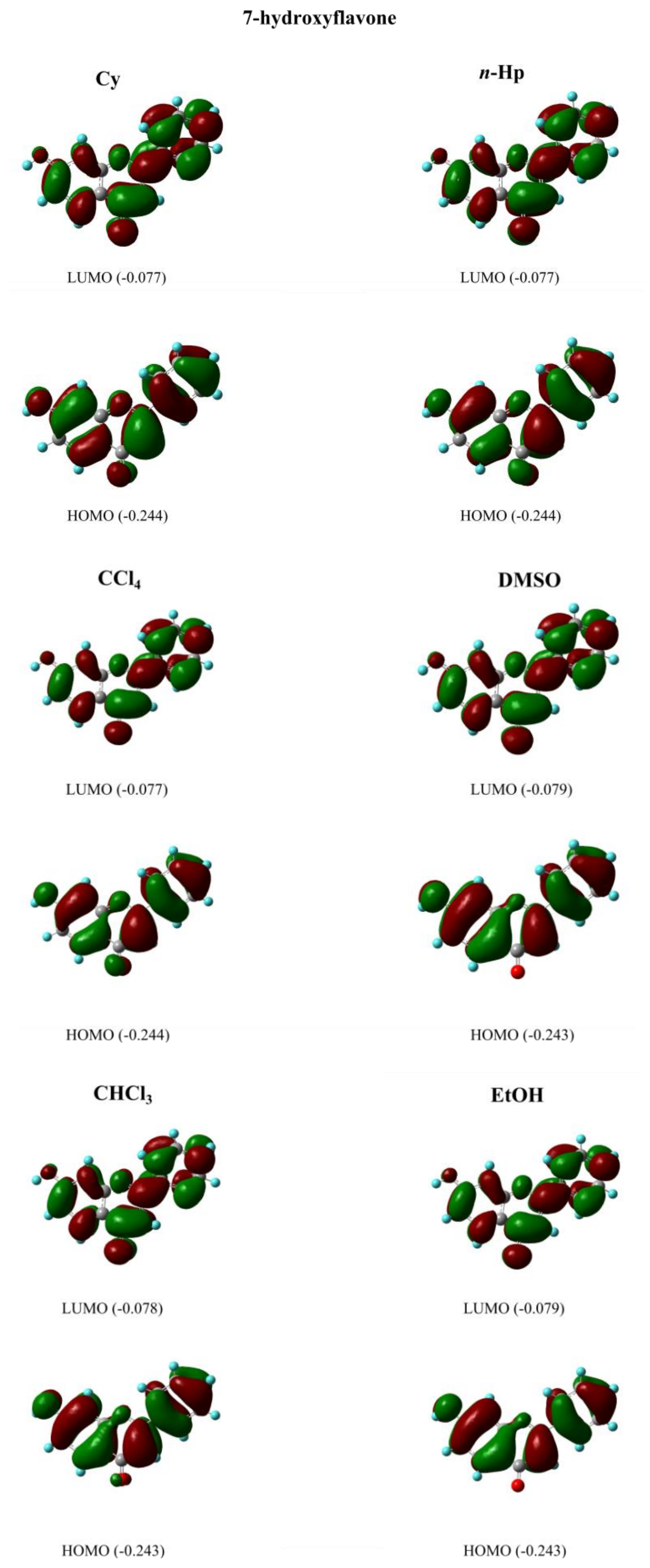

Figure S3. Computer Visualization of the Molecular Orbitals Involved in the Electronic Transitions of Flavone in Different Solvents Calculated with the TD-PBE0/6-311+G(2d,2p) Method and the IEF-PCM Model.

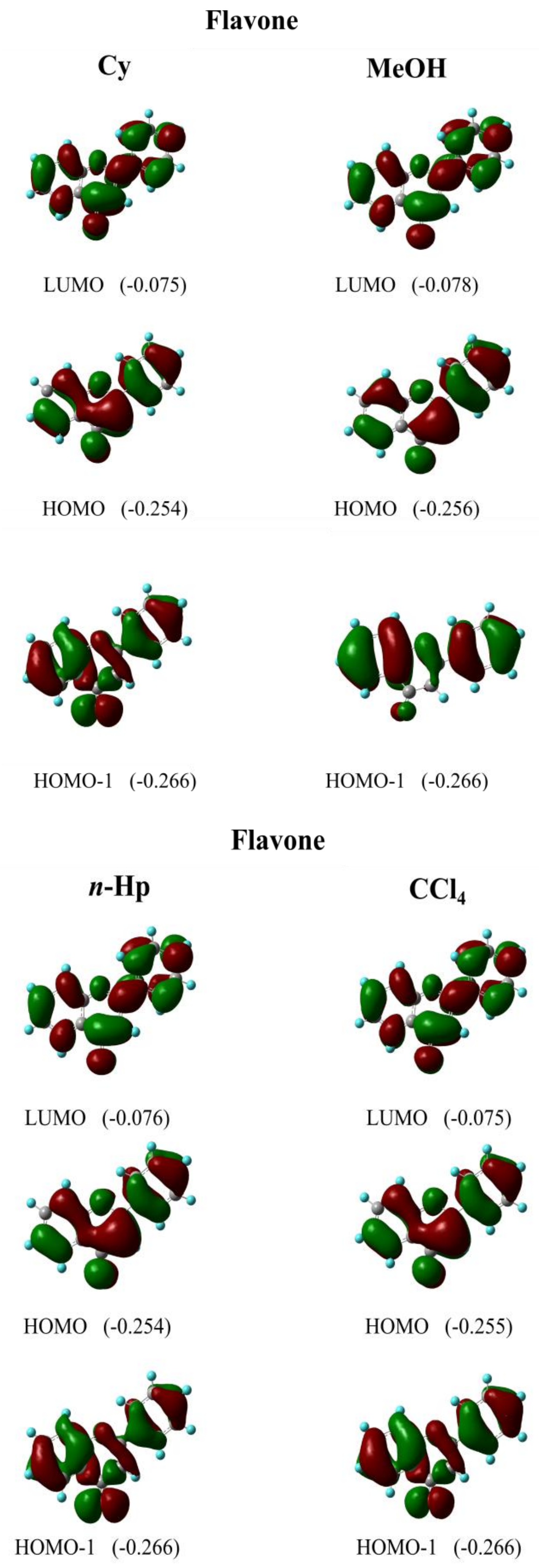

Figure S3. *Cont.*

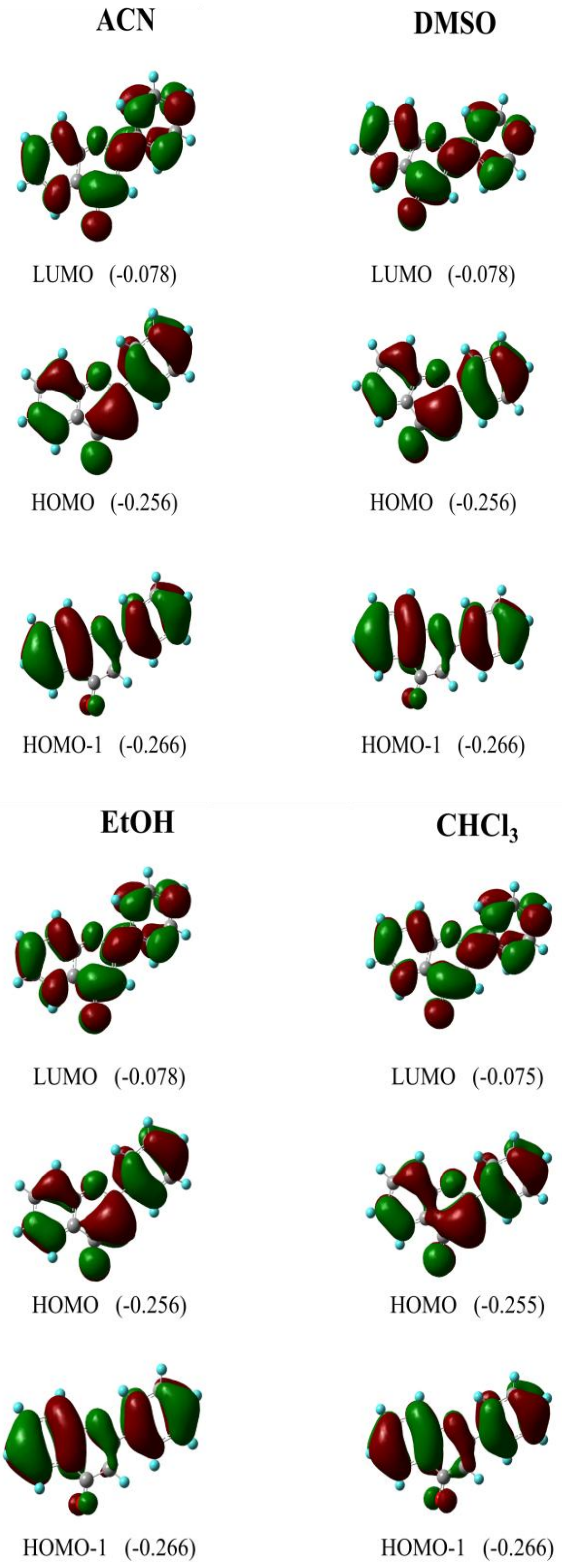

Figure S4. Computer Visualization of the Molecular Orbitals Involved in the Electronic Transitions of 7-hydroxyflavone in Different Solvents Calculated with the TD-PBE0/6-311+G(2d,2p) Method and the IEF-PCM Model.

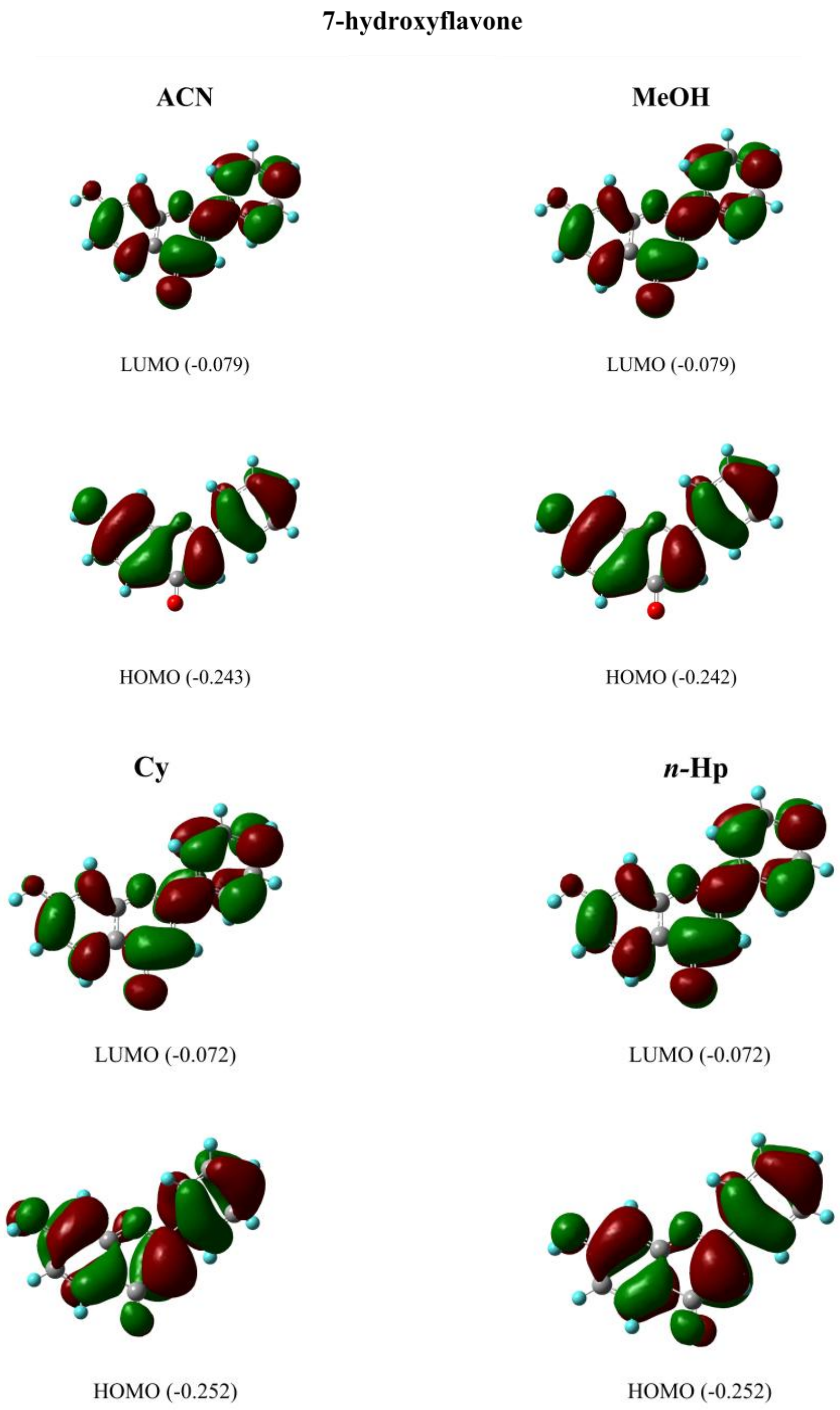

Figure S4. *Cont.*

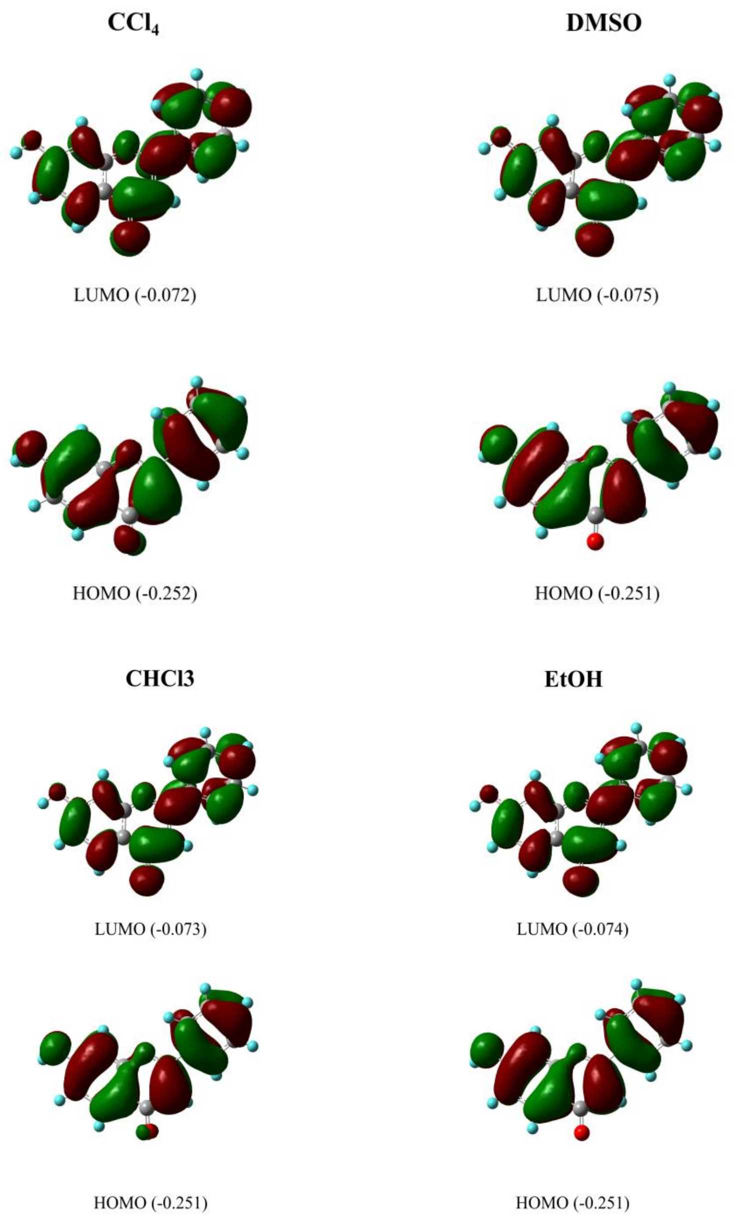

Supplement: Supplementary file 1 [file ijms-12-08895-s001.pdf]
